# An Overview of SARS-CoV-2 and Animal Infection

**DOI:** 10.3389/fvets.2020.596391

**Published:** 2020-12-11

**Authors:** Mohamed A. A. Mahdy, Waleed Younis, Zamzam Ewaida

**Affiliations:** ^1^Department of Anatomy and Embryology, Faculty of Veterinary Medicine, South Valley University, Qena, Egypt; ^2^Department of Microbiology, Faculty of Veterinary Medicine, South Valley University, Qena, Egypt; ^3^Qena University Hospital, South Valley University, Qena, Egypt

**Keywords:** animal, animal model, COVID-19, Intermediate host, SARS-CoV-2, zoonosis

## Abstract

A novel coronavirus has been reported as the causative pathogen of the Coronavirus disease 2019 (COVID-19) outbreak in Wuhan city, China in December 2019. Due to the rapid spread of the virus worldwide, it has been announced as a pandemic by the World Health Organization (WHO). Hospitalized patients in Wuhan were associated with the Huanan seafood wholesale market where live animals, such as poultry, bats, snakes, frogs, rabbits, marmots, and hedgehogs are sold in that market which suggests a possible zoonotic infection. It was suggested that bat is the natural host of SARS-CoV-2, but the intermediate host is still unclear. It is essential to identify the potential intermediate host to interrupt the transmission chain of the virus. Pangolin is a highly suspected candidate as an intermediate host for SARS-CoV-2. Recently, SARS-CoV-2 infection has been reported in cats, dogs, tigers, and lions. More recently SARS-CoV-2 infection affected minks severely and zoonotic transfer with a variant SARS-CoV-2 strain evidenced in Denmark, Netherlands, USA, and Spain suggesting animal-to-human and animal-to-animal transmission within mink farms. Furthermore, experimental studies documented the susceptibility of different animal species to SARS-CoV-2, such as mice, golden hamsters, cats, ferrets, non-human primates, and treeshrews. It is also essential to know the possibility of infection for other animal species. This short review aims to provide an overview on the relation between severe acute respiratory syndrome coronavirus-2 (SARS-CoV-2) infection and animals.

## Introduction

Several pneumonia cases with an unknown cause have been reported in Wuhan city, the capital of Hubei province, China on December 31st, 2019 (1, 2). The disease was characterized by a respiratory disorder of variable degree of severity ranged from mild upper respiratory tract illness to acute respiratory distress syndrome and severe interstitial pneumonia (3). On January 7th, 2020, a novel coronavirus (nCoV-2019) has been isolated and identified as the causative pathogen of the coronavirus disease (COVID-19) (4). Later, the International Committee on Taxonomy of Viruses has named the virus as severe acute respiratory syndrome coronavirus-2 (SARS-CoV-2) (5). Due to the rapid spread of the virus in many countries worldwide, the World Health Organization (WHO) announced it as a pandemic on March 11th, 2020 (4). The confirmed cases are 56,623,643 and mortalities are 1,355,963 in more than 220 countries, as of November 20th, 2020 (4).

Coronaviruses (CoVs) are enveloped +ve-sense, single-stranded RNA viruses classified within the subfamily Coronavirinae, family Coronaviridae (6). The name coronavirus comes from its appearance under electron microscopy as a crown-like structure (7). CoVs have four genera alpha (α), beta (β), gamma (γ), and delta (δ) coronaviruses that primarily originate from animals (8, 9). Mammals, specifically bats, are the natural hosts of α- and β-CoVs, while pigs and birds are the natural hosts of γ- and δ-CoVs (10). CoVs have a great ability to mutate which facilitates their transmission from animals to humans (11). Crossing the species barrier to infect humans results in outbreaks (7).

SARS-CoV-2 has been classified as a novel member of the β-coronavirus genus by the whole genome sequencing. It belongs to the subgenus sarbecovirus of the Coronaviridae family. SARS-CoV-2 has RNA genome of about 30 kb (12) that encodes 16 non-structural proteins and four structural proteins, including spike (S), envelope (E), membrane (M), and nucleocapsid (N) proteins (13, 14). The S protein of CoVs constitutes the spike on the virion surface, it gives the virion crown-like appearance and plays a vital role in host range determination, recognition of host receptors, viral binding, fusion, entry, and tissue tropism, as well as, the induction of neutralizing antibody and T cell responses (14–16). The M protein is responsible for the specific shape of the viral envelope, it forms ribonucleoproteins and mediates inflammatory reactions in host cells. The E protein is a membrane polypeptide that acts as an ion channel (viroporin), it promotes viral pathogenicity while N protein helps viral entry and viral survival in host cells (16).

SARS-CoV-2 was firstly identified in hospitalized patients in Wuhan city, Hubei Province, China in December 2019 (2). Those patients were associated with the Huanan seafood wholesale market where live animals, such as poultry, bats, snakes, frogs, rabbits, marmots, and hedgehogs are sold in that market suggesting a possible zoonotic spillover (17, 18). It was suggested that SARS-CoV-2 originated from bats (19). Different animal species, such as snake, pangolin, and turtle were suggested as potential intermediate hosts, however, pangolin was the highly suspected candidate as its intermediate host (20–22). Moreover, several animal species have been reported to be susceptible to SARS-CoV-2 infection either naturally (cats, dogs, minks, lions, tigers) or after experimental infection (mice, cats, ferrets, hamsters, primates, treeshrew) (13, 23–32). Also, the high mutation rates of RNA viruses help them to adapt to a wide range of hosts (33). Therefore, it is essential to identify the potential virus reservoir and the possibility of infection for other animal species. This review aims to provide an overview of the relation between SARS-CoV-2 and animals.

## Possible Animal Reservoirs

It is assumed that SARS-CoV-2 has been originated from animals and transmitted to humans, then maintained human-to-human transmission (21). Several animal species have been reported to be susceptible to SARS-CoV-2 infection. The variety of species susceptible to SARS-CoV-2 infection indicates that the virus crosses the species barrier (13). Therefore, many animals either wild or domestic may be infected and act as intermediate hosts for SARS-CoV-2 virus (18, 20). Anderson and his colleagues proposed two possible scenarios for the origin of SARS-CoV-2; either the virus underwent natural selection in an animal host before its transmission to humans or the virus underwent natural selection in humans after being transmitted to humans (34).

**Figure 1:**
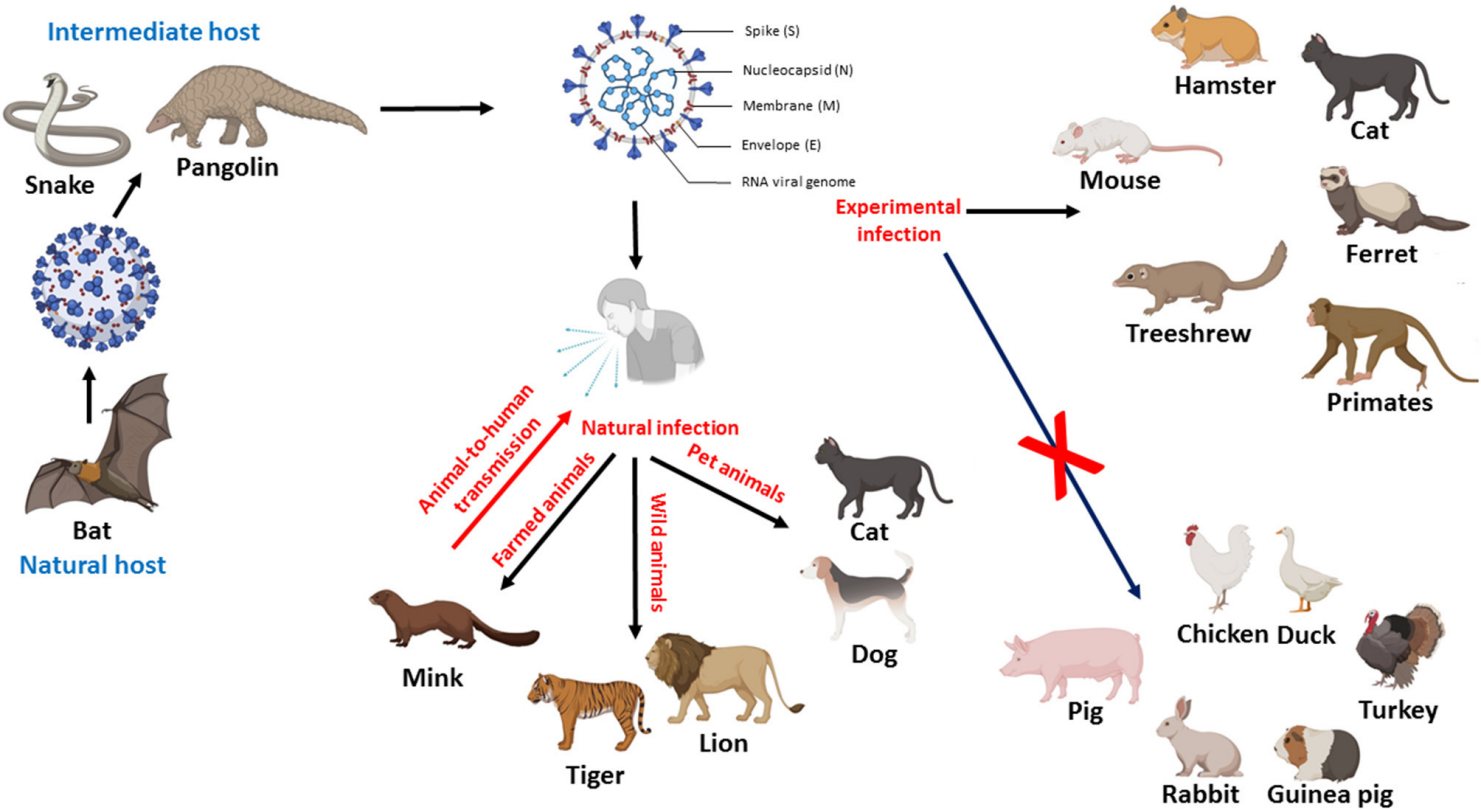
Diagram showing the possible role of animals in the transmission of SARS-CoV-2, potential intermediate hosts, natural and experimental infection of animals. Figure was created with BioRender.com.

It was suggested that bat is the natural host of SARS-CoV-2, but the intermediate host is still unclear (19). It is essential to identify the potential intermediate host to interrupt the transmission chain of the virus. Pangolin is a highly suspected candidate as an intermediate host for SARS-CoV-2 (20). Recently, SARS-CoV-2 infection has been reported in cats, dogs, minks, tigers, and lions (28, 31, 35–37). Furthermore, experimental studies documented the susceptibility of different animal species to SARS-CoV-2, such as mice, hamsters, cats, ferrets, non-human primates, and treeshrews (6, 24, 30, 38, 39). The natural and experimental infection to animals is shown in Figure 1.

### Bats

It has been reported that bats are the main natural reservoir host of several CoVs. Among the 1100 different species of bats, the horseshoe bat [*genus Rhinolophus (R.)*] is the reservoir of the SARS-like-CoVs (40). Both SARS and Middle East respiratory syndrome (MERS) CoVs originated from bats and passed through the intermediate hosts palm civets and camels, respectively (12). Therefore, it was suggested that bat might be the potential origin of SARS-CoV-2. It has been shown that SARS-CoV-2 was about 96.2% identical to the genetic information of the bat (*R. affinis*) SARS-like CoV (RaTG13) based on genome sequencing and evolutionary analysis (2, 21, 41, 42). The similarity between SARS-CoV-2 and bat coronavirus suggests the possibility that SARS-CoV-2 originated from bats (41–44) like SARS-CoV and MERS-CoV (7). It was hypothesized that at least two bat species, *R. affinis* and *R. malaynus*, might be the proposed natural hosts of SARS-CoV-2 virus (19). Due to the lack of direct contact between bats and humans, direct human infection with bat CoVs is rare. It was assumed that transmission of SARS-CoV-2 from bats to human passes through an unknown intermediate host that might facilitate its transfer to humans (21, 41–43). The analysis of genomic sequences of CoVs suggested that SARS-CoV-2 was a recombinant virus that arises between the bat CoV and another coronavirus of unknown origin (21). Meanwhile, a mutation in S Glycoprotein and N protein of SARS-CoV-2 makes it distinct from bat SARS-like CoV supporting the hypothesis that SARS-CoV-2 mutated before its transmission from bats and acquired its ability to infect human (42). SARS-CoV-2 invades cells mainly through binding of the virus S protein and host cell receptor, angiotensin-converting enzyme 2 (ACE2) (45, 46). Although, SARS-CoV-2 recognizes ACE2 from a variety of animal species, including palm civet, SARS-CoV-2 acquires the capability to infect humans, as well as, to transmit among humans (46). The binding affinity of SARS-CoV-2 to human ACE2 is higher than that of SARS-CoV (45, 46) which suggests a possibility of cross-species transmission (47) and the rapid spread of this virus (45, 46). On the other hand, experimentally infected Egyptian fruit bats showed no symptoms of the disease, moreover, they did not infect other bats (48).

### Pangolins

The whole genome of pangolin-CoV isolated from Malayan pangolin (*Manis javanica*) was highly similar to that of the SARS-CoV-2 and bat CoV (49). But, the similarity between pangolin-CoV and SARS-CoV-2 was higher than that with the bat CoV (50, 51). Moreover, pangolin CoVs shared 5 identical amino acids with SARS-CoV-2 whereas bat CoV RaTG13 only shared one amino acid with SARS-CoV-2 suggesting that pangolin could be a potential intermediate host that might mediate the cross-species transmission of SARS-CoV-2 (20, 52). Furthermore, the receptor-binding domain (RBD) of the pangolin-CoV was nearly identical to that of SARS-CoV-2 with a strong binding ability to human ACE2 indicating that pangolin was involved in the recombination of SARS-CoV-2 (49). However, the phylogenetic analyses support that SARS-CoV-2 did not arise directly from the pangolin-CoV (52, 53). Therefore, it was suggested that SARS-CoV-2 originated in bats and transmitted to pangolin where recombination of pangolin-CoV and bat-CoV-RaTG13-like virus occurred. Then the recombined virus gets the ability to infect human cells (12, 20).

### Reptiles

Analysis of the structural binding mechanism of SARS-CoV-2 RBD and ACE2 receptors revealed that turtles (*Chrysemys picta bellii, Pelodiscus sinensis, and Chelonia mydas*) and snakes might act as one of the potential intermediate hosts that transmit SARS-CoV-2 to humans (22, 54). Moreover, evolutionary analysis, as well as analysis of the codon usage of SARS-CoV-2 suggested that snakes might be a potential wildlife animal reservoir for SARS-CoV-2 (21, 22). However, ACE2 of turtle and snake lost its capability to bind to S protein of SARS-CoV-2, therefore these reptiles should not be considered as potential hosts for SARS-CoV-2 (55).

### Other Animals

Structural analysis of the binding mechanism of RBD of SARS-CoV-2 and ACE2 receptors suggests that ACE2 from fish, amphibians, birds, and mammals can bind to RBD of SARS-CoV-2 making them possible natural hosts for SARS-CoV-2 (54). A recent study examined serum samples from 35 different animal species to detect SARS-CoV-2-specific antibodies using SARS-CoV-2 ELISA kit (56). The serum samples were collected from poultry (chicken, duck, and goose), experimental animals (mice, rat, and rhesus monkey), companion animals (cat and dog), domestic animals (sheep, pig, horse, and cow), wild animals (leopard cat, masked civet, mink, ferret, jackal, fox, alpaca, camel, eagle, bamboo rat, peacock, tiger rhinoceros, porcupine, bear, giant panda, red pandas, pangolin, weasel, yellow-throated marten, and wild boar). All serum samples examined had no SARS-CoV-2-specific antibodies which excluded the possibility of these animal species as intermediate hosts for SARS-CoV-2 (56).

## SARS-CoV-2 and Animal Infection

The hospitalized patients in Wuhan were associated with the Huanan seafood wholesale market. In this market, live animals, such as poultry, bats, snakes, frogs, rabbits, marmots, and hedgehogs are sold for human consumption suggesting a possible zoonotic infection (17). It is critical to understand the susceptibility of animals to SARS-CoV-2 to control the spread of the virus. Until now, several cases of human-to-animal transmission during the pandemic have been reported in several countries worldwide, including Hong Kong, Belgium, Germany, United States, Spain, Netherlands, Denmark, and France (23, 31, 57). Although there is no evidence that animals had a role in the spread of the SARS-CoV-2 pandemic (58), there is a raising alarm that animals may get infected and transmit the virus to humans (37).

### Cats

SARS-CoV-2 infects cat populations in Wuhan during the COVID-19 outbreak based on detection of SARS-CoV-2 specific antibodies in 15/102 (14.7%) of sampled cats. Infection to those cats raised under natural condition upon contact with SARS-CoV-2 infected patients or SARS-CoV-2 polluted environment and developed antibody response (32). The later author added that a higher antibody titer was detected in cats that were living in close contact with SARS-CoV-2 infected owners. Moreover, the U.S. Centers for Disease Control and Prevention (CDC) announced SARS-CoV-2 infection in two pet cats for the first time in two separate places in New York. Both cats were tested after showing mild respiratory symptoms. One of them might receive infection from her infected owner, while the other cat might receive infection either from asymptomatic household members or upon contact with an infected individual outside its home (35, 57). Also, SARS-CoV-2 was detected in the feces and vomit of two infected pet cat living with infected owners in Belgium and Hong Kong indicating active replication of the virus (13, 59, 60). In this respect, SARS-CoV-2 has been reported to replicate only in the upper respiratory tract of cats and this replication was not associated with severe disease or death (30). It is worth to mention that younger cats were more tolerant to SARS-CoV-2 infection (30). Moreover, cats can transmit the infection to other cats (61). Therefore, pet cats are more susceptible to SARS-CoV-2 than dogs, but with mild symptoms and virus shedding (62).

### Dogs

Although dogs have low susceptibility to SARS-CoV-2 infection (30), two pet dogs from Hong Kong and another from North Italy were infected with SARS-CoV-2 infection, without symptoms, due to contact with SARS-CoV-2 infected persons (13, 27, 31, 47, 60). Dogs have ACE2 receptors, similar to human ACE2 (hACE2), that function as SARS-CoV receptors which raises the possibility that dogs might be a potential intermediate host (47). Although there is no evidence that infected dogs can transmit the virus to either animals or humans (31). Recently, Freuling et al. (63) showed the susceptibility of raccoon dogs to SARS-CoV-2 infection following intranasal inoculation. Virus shedding was detected in nasal and oropharyngeal swabs of infected dogs at 2nd day post-infection (dpi). Moreover, infected dogs were capable to transmit the virus to contact animals which suggested that raccoon dogs might be a potential reservoir for SARS-CoV-2 (63).

### Minks

Minks are the first intensively farmed species to be affected by the COVID-19 outbreak indicating a higher susceptibility of mustelids to SARS-CoV-2 (64). Several mink farms have been attacked by SARS-CoV-2 at first in the Netherlands, then in Denmark, USA, and Spain (23, 65, 66). It was suspected that viral infection was transmitted from an infected farm worker to minks (28). Infected minks developed signs of respiratory disease ranging from watery nasal exudate to severe respiratory distress together with gastrointestinal disorders (23, 62, 67). Minks presented moderate to severe signs were found dead, and the necropsy finding showed severe pneumonia (67). The histopathological picture included severe diffuse interstitial pneumonia, alveolar damage, pulmonary edema, and inflammatory cellular infiltration. Viral antigen was detected in nasal conchae, trachea, and epithelial cells, while the viral RNA was detected in nasal conchae, throat swabs, lung, and rectal swabs (28, 67). Genetic and epidemiological studies reported infection of farm workers after the outbreak in mink farms indicating animal-to-human and animal-to-animal transmission within mink farms (68, 69). It is worth to mention that SARS-CoV-2 isolated from mink (MT396266) was highly similar to human SARS-CoV-2 (44). There are arising worries of spreading of SARS-CoV-2 among wild mustelids as they might become permanent reservoirs of the virus (64).

### Other Animals

A Malayan tiger at Bronx Zoo, New York, USA was tested positive for SARS-CoV-2 as the first case of animal infection in USA. This tiger was the first infected tiger anywhere in the world and the first case of human to non-domestic animal transmission (36, 60). Later, the infection has been detected in four tigers and three lions (37, 70) indicating that different feline species are susceptible to SARS-CoV-2 infection (60). It was suspected that the tiger received infection from an infected asymptomatic employee (13, 36, 70). On the other hand, Ulrich et al. (71) reported that cattle had low susceptibility to SARS-CoV-2 infection. While pigs and several poultry species including chickens, turkeys, ducks, geese, and Japanese quail were not susceptible to SARS-CoV-2 infection (30, 48, 72, 73). Furthermore, rabbit and guinea pig housed with COVID-19 patients were tested negative for SARS-CoV-2 infection (74).

## Animal Models for Studying SARS-CoV-2

There is an urgent need for animal models that mimic the clinical and pathological characteristics of human SARS-CoV-2 infection to help to study its pathogenesis. These models will help to assess the efficacy of vaccine trials, proper dose, route of vaccine delivery. In addition to assessing the therapeutic potential and safety of the novel antiviral treatment (24, 75–77). The ideal animal model should presents similar clinical characteristics to the disease in humans, such as the active infection, clinical symptoms in humans, virus shedding, transmission to naïve animals, and host immune response (26, 75, 76). The available animal models for SARS-CoV-2 are listed in Table 1.

**Table 1:** The available animal models for SARS-CoV-2.

	**Animal model**	**Clinical symptoms**	**Histopathological features**	**Viral titer**	**Advantages**	**Disadvantages**	**References**
Mice	- Wild type mice	- No weight loss		- Low level of viral RNA at 10 dpi		- Inefficient virus replication	(78)
	- hACE2-transduced mice	- Weight loss in the first week	- Extensive neutrophil infiltration in lung alveoli and around blood vessels	- High viral titers in the lung tissue	- Easily reproducible using adenovirus vector in commercial mouse strains - Can be generated within short time.	- Variation in hACE2 expression from mouse-to-mouse - Mild bronchial inflammation associated with delivery of Adenovirus	(78, 79)
	- Transgenic mice expressing hACE2	- Weight loss in aged mice only	- Lymphocytes and monocytes infiltration in the alveolar interstitium in young and aged transgenic mice	- Viral replication in lungs of young and aged transgenic mice - Viral RNA in feces of aged mice only	- High susceptibility of hACE2 transgenic mice to intranasal infection of SARS-CoV-2 - This model mimics the mild cases of COVID-19	- Do not develop severe disease. - No extrapulmonary picture of disease - Limited availability of the transgenic mice and the high costs	(6, 80)
	- Mouse-adapted SARS-CoV-2 strain (MASCp6) in aged BALB/c mice	- Mild to moderate pneumonia - No significant weight loss	- Interstitial pneumonia with thickened alveolar septa, damage alveoli - Focal hemorrhage and exudate - Injured blood vessels.	- Viral replication in the trachea and lung of young and aged BALB/c mice	- Resemble lung damage and inflammatory responses in COVID-19 patients - Economical, convenient, and effective model to evaluate vaccines and therapeutics	- Do not develop severe disease.	(81)
Hamster	- Syrian Golden hamster	- Progressive weight loss - Rapid breathing - Lethargy, hunched back, and ruffled furs	- Focal inflammation in the lung - Diffuse alveolar destruction - Pulmonary edema, and alveolar hemorrhage - Mononuclear inflammatory cell infiltration - Lung consolidation	- High level of virus titer in the nasal turbinates, trachea, and lungs at 2–7 dpi	- Easy to handle - High susceptibility to infection due to high binding affinity of ACE2 protein to the Spike of SARS-CoV-2 - Develop severe pneumonia similar to human patients - Infected hamsters develop immunity against reinfection		(24, 82, 83)
	- Chinese hamster	- Has a similar course of disease as in Syrian hamster - Milder and prolonged pneumonia than in Syrian hamster	- Has a similar histopathological picture of the disease as in Syrian hamster	- The same as Syrian hamser	- Highly susceptible to SARS-CoV-2 infection - More suitable than Syrian hamster due to its smaller size		(84)
- Cats		- Experimentally infected cats are asymptomatic	- Massive lesions in the nasal and tracheal epithelium, and lungs	- Viral RNA in the nasal turbinate, trachea, lung, and small intestine	- Easy transmission between cats		(30, 85)
- Ferrets		- Fever - Loss of appetite - Occasional coughs - Reduced activity	- Severe lymphoplasmacytic perivasculitis and vasculitis - Mild peribronchitis in the lungs, - Inflammatory cell infiltration in the alveolar septa and lumen	- Viral RNA in the nasal turbinate, tonsils, and soft palate - Virus shedding urine and fecal samples	- High susceptibility to SARS-CoV-2 infection - Active transmission from infected ferrets to naïve ones through direct contact and occasionally airborne transmission	- Mild symptoms - Low virus titer in lungs	(26, 30)
Primates	Cynomolgus macaques	- Asymptomatic except serous nasal discharge in an aged animal	- Focal pulmonary consolidation in young and aged animals - Edema in alveolar and bronchiolar lumina - Thickened alveolar walls - Hyaline membrane formation - Hyperplasia of type II pneumocyte, and mononuclear infiltration	- Viral replication in both upper and lower respiratory tracts. - Viral replication peaks at the early stages of infection. - Higher level of viral RNA expression and prolonged virus shedding in aged animals compared to young animals	- Effective virus transmission to other animals - Development of lung disease - Early peak of virus resembles asymptomatic patients	- Slower reproduction rate - Limited clinical signs developed - High cost - Difficult handling - Ethical reasons	(38, 86)
	Rhesus macaques	- Asymptomatic or show mild and transient symptoms, such as, reduced appetite, weight loss, elevated body temperature, rapid respiration, hunched posture, dehydration, pale appearance, and occasional coughing	- Lung consolidation, edema, hemorrhage - Thickened alveolar walls, inflammatory infiltration - Mild to moderate interstitial pneumonia - Old animals shows diffuse severe interstitial pneumonia	- Viral RNA is in pharynx, trachea, bronchi, and lungs - High level of virus shedding from the nose and throat	- ACE2 receptors are 100% identical to those of humans - The moderate and transient disease resemble that of human cases - Similar virus shedding pattern to that of human	- Slower reproduction rate - Moderate clinical signs developed - High cost - Difficult handling [-] Ethical reasons	(29, 87–90)
- Treeshrew		- Asymptomatic with elevated temperature in female animals	- Mild histopathological picture in lung tissue including local hemorrhagic necrosis, lung consolidation at the margin, and inflammatory infiltration	- Low level of viral shedding with earlier shedding in young animals	- Alternative to primates	- Limited virus replication - Mild histopathology	(39)

### Rodents

#### Mice

Mouse models are useful tools to evaluate vaccines and antiviral therapeutics. Although several mouse models have been described as potential models for COVID-19, none of these models recapitulated all characteristics of COVID-19 in humans (91).

SARS-CoV-2 has a very low binding affinity to ACE2 of commercially available mice strains compared with that in humans. Experimentally infected laboratory mice neither showed weight loss nor a high level of viral RNA at day 10 post-infection indicating inefficient replication of the virus (78). Therefore, laboratory mice cannot be used as animal models to test vaccine or antiviral drugs (78, 92). Delivery of hACE2 receptor to commercially available mice using adenovirus vector enhanced their susceptibility to SARS-CoV-2 lung infection, clinical disease, and pathology (78, 79). Infected hACE2-transduced mice showed weight loss, hunching, ruffled fur, and difficulty breathing at 2 dpi. The gross lesions included vascular congestion and hemorrhage at 5 dpi (79) with detection of high viral titer in the lung tissue together with extensive immune cellular infiltration, mostly neutrophils, in lung alveoli and around blood vessels with alveolar edema and necrotic debris (78, 79). These findings indicated that the viral infection is localized entirely in the lungs. It is worth to mention that recovered mice developed protective immunity against reinfection (25). Development of SARS-CoV-2-susceptible mouse model using adenovirus vector in commercially available strains is an easily reproducible murine model for SARS-CoV-2 within a short time, which may hasten the process of virus identification, and vaccine development.

The experimentally infected transgenic mice expressing hACE2 receptors showed no clear symptoms, except weight loss in aged mice only (6, 80). Virus replication was detected in trachea, lungs, and brain together with lymphocytes and monocytes infiltration in the alveolar interstitium in both young and aged transgenic mice (6) with viral RNA detection in feces of aged mice only (6, 80). This indicated the high susceptibility of hACE2 transgenic mouse model intranasal infection of SARS-CoV-2 and this model resembled the mild cases of COVID-19, but not the severe and lethal cases (6, 80). However, the very limited availability of these transgenic mice together with the high costs are the major limitations of this model.

Gu et al. (81) generated a mouse-adapted SARS-CoV-2 strain, MASCp6, by serial passage of a SARS-CoV-2 clinical isolate in aged BALB/c mice. The infected young and aged mice developed mild to moderate pneumonia with no significant weight loss. A stable viral replication in the trachea and lung of young, and aged BALB/c mice was observed. Aged mice expressed interstitial pneumonia with thickened alveolar septa, damaged alveoli, focal hemorrhage and exudate, and injured blood vessels. The lung damage and inflammatory responses in this model resembled those in COVID-19 patients. Therefore, this animal model could be an economic, convenient, and effective mouse model to evaluate vaccines and therapeutics against SARS-CoV-2 infection (81).

#### Hamsters

Golden Syrian hamster (*Mesocricetus auratus*) is considered as an excellent model supporting the replication of SARS-CoV infection (93). Both the clinical and histopathological findings of the SARS-CoV-2-infected hamster closely resembled the manifestations of human upper and lower respiratory tract infection with virus shedding in respiratory droplets and feces. Infected hamsters showed progressive weight loss, rapid breathing, lethargy, hunched back, and ruffled furs (24, 82). Micro-CT imaging showed severe lung abnormalities, such as peribronchial ground-glass opacity at 2 dpi that converted into multilobular ground-glass opacity with patches of lung consolidation at 7–8 dpi (83). The histopathological findings included focal inflammation in the lung, diffuse alveolar destruction, pulmonary edema, alveolar hemorrhage, mononuclear inflammatory cell infiltration, and alveolar collapse at 2–3 dpi. Lung consolidation increased at 4–6 dpi with severe pulmonary hemorrhage. Virus titer was detected at a high level in the nasal turbinates, trachea, and lungs at 2–7 dpi (24, 82, 83, 94). On the other hand, the virus was transmitted to naïve hamsters in close contact with infected ones and via aerosol (24, 82). Furthermore, the Syrian hamster ACE2 protein showed a high binding affinity to the Spike protein of SARS-CoV-2 (24, 95). It is worth to mention that infected hamsters developed immunity against reinfection (83). Taken together, the Syrian hamster is highly susceptible to SARS-CoV-2 and it could be a suitable model simulating the clinical, pathological, virological, and immunological features of SARS-CoV-2-infection (24, 83, 95).

Chinese hamsters (*Cricetulus griseus*) are highly susceptible to SARS-CoV-2 infection, they showed a similar course of disease and lung histopathology to that reported in Syrian hamsters (84). However, Chinese hamsters showed milder and prolonged pneumonia than that reported in Syrian hamsters indicating slower recovery than Chinese hamsters. The considerably smaller size of the Chinese hamster than the Syrian hamster makes it a more suitable animal model to study SARS-CoV-2 (84).

### Cats

Experimentally infected cats with SARS-CoV-2 were asymptomatic but expressed viral RNA in the nasal turbinate, trachea, lung, and small intestine indicating that the virus replicated well in the upper respiratory tract of those cats (30, 96). Viral RNA was also detected in tonsils, lymph nodes, spleen, bone marrow, liver, kidney, heart, and olfactory bulb (96). The histopathological features in juvenile cats included massive lesions in the epithelium of the nose and trachea, and lungs indicating efficient replication of the virus in younger cats than older ones (30). Also, multifocal lymphocytic and neutrophilic infiltration were reported in the lamina propria and submucosa of the trachea and bronchi (96). Moreover, SARS-CoV-2 was transmitted from an infected cat to a non-infected one through direct contact with respiratory droplets (30, 85). Infected cats with no symptoms might act as a silent intermediate host of SARS-CoV-2 (85).

An infected pet cat with SARS-CoV-2 in Belgium showed clinical signs, such as obvious lethargy, anorexia, poor appetite, vomiting, and diarrhea. Later, the cat showed sneezing, productive cough, difficult breathing, and emaciation. Moreover, viral RNA persisted for about 10 days with virus shedding in the feces (59). Therefore, cats also could be used as an optimal animal model for asymptomatic-to-moderate COVID-19 and screening antiviral drugs or vaccines against SARS-CoV-2 (91, 92). However, it is difficult to handle cats in biosafety level-3 conditions (91).

### Ferrets

Ferrets are frequently used as an animal model for studying human respiratory viral infection (97, 98). They were highly susceptible to infection with SARS-CoV-2 (26, 48, 99). Experimentally infected ferrets showed fever, loss of appetite, occasional coughs, and reduced activity (26, 30). The histopathological features included severe lymphoplasmacytic perivasculitis and vasculitis, mild peribronchitis in the lungs, and inflammatory cell infiltration in the alveolar septa and lumen. Viral RNA was detected in the nasal turbinate, tonsils, and soft palate indicating virus replication in the upper respiratory tract (26, 30, 94, 99). Additionally, virus shedding was reported in urine and fecal samples (26).

SARS-CoV-2 was actively transmitted from infected ferrets to naïve ones through direct contact and occasionally airborne transmission (26, 48). Infected ferrets were capable to transmit the virus at 2 dpi before reaching the peak of viral RNA copy number and the peak of body temperature as well (26, 99). Therefore, ferret is considered as the most closely animal model mimicking human infection and transmission (99). However, infected ferrets showed some limitations, such as mild symptoms and low virus titer in the lungs (26). Recently, ferret was used as an animal model to evaluate the efficacy of three FDA-approved drugs, lopinavir-ritonavir, hydroxychloroquine sulfate, and emtricitabine-tenofovir, against SARS-CoV-2 infection (100).

### Primates

The cynomolgus macaques is an animal model that is closest to humans in pathophysiology, therefore, it can be used for SARS-CoV-2 studies (38). Infected young and aged macaques were asymptomatic except for serous nasal discharge in an aged animal. The pathological features included focal pulmonary consolidation in both young and aged animals; edema in alveolar and bronchiolar lumina, thickened alveolar walls, hyaline membrane formation, hyperplasia of type II pneumocyte, and mononuclear infiltration (38, 101). Viral replication was detected in both the upper and lower respiratory tracts, which corresponds to the effective virus transmission to other animals and the development of lung disease, respectively. Moreover, viral replication peaked at the early stages of infection that resembled asymptomatic patients. On the other hand, aged animals expressed a higher level of viral RNA and prolonged virus shedding compared to young animals (101).

The infected rhesus macaques were also asymptomatic or showed mild and transient symptoms, such as reduced appetite, weight loss, elevated body temperature, rapid respiration, hunched posture, dehydration, pale appearance, and occasional coughing (29, 87–90). The chest X-ray showed patchy to mild glass-ground opacity in the lower parts of lungs up to 3 dpi, the density decreased at 6 dpi (29). The pathological features included variable degrees of lung consolidation, edema, hemorrhage, thickened alveolar walls, inflammatory infiltration, and mild to moderate interstitial pneumonia (29, 87, 89, 90). Infected old rhesus macaque showed diffuse severe interstitial pneumonia with extremely thickened alveolar septa than in young rhesus macaque (90, 102). Viral RNA was detected in the pharynx, trachea, bronchi, and lungs with a high level of virus shedding from the nose and throat (87–89). It is worth to mention that primarily infected monkeys developed immunity against SARS-CoV-2, which protected those monkeys from the second infection (87). It is worth to mention that the ACE2 receptors of rhesus macaques were about 100% identical to those of humans (76). Additionally, the moderate and transient disease developed in rhesus macaque was similar to that of the majority of human cases together with a similar virus shedding pattern to that of human (88). Therefore, rhesus macaque could be a suitable animal model that mimics mild or asymptomatic human cases and to evaluate potential drugs and vaccine safety (29, 88, 102). However, the slower reproduction rate of cynomolgus and rhesus macaques is considered a major limitation of using these animals (38).

In a recent study, Lu et al. (102) experimentally infected three types of non-human primates; rhesus macaques *(Macaca mulatta)* and cynomolgus macaques (*Macaca fascicularis)* as old world monkeys and common marmosets *(Callithrix jacchus)* as new world monkeys with SARS-CoV-2. They concluded that the three non-human primates simulated several features of COVID-19 and that *M. mulatta* was the most susceptible to SARS-CoV-2 infection, while *C. jacchus* was the least susceptible to infection (102). Even though non-human primates are closely similar to human beings, which make them ideal candidates for vaccine evaluation (76), their main disadvantages are the high cost, difficult handling, and ethical reasons (62, 103).

### Treeshrew

The treeshrew (*Tupaia belangeris*) is a non-rodent, primate-like animal. It has been used in biomedical research as an animal model for viral infections (104). Treeshrew inoculated with SARS-CoV-2 showed no clinical signs with limited replication of the virus and mild histopathological abnormalities in lung tissue. Therefore, treeshrew is not as a susceptible model to SARS-CoV-2 infection (39).

## Prevention and Control of COVID-19

### Precautions on Pet Animals

Although there is no confirmation that pet animals have a role in the spreading of the SARS-CoV-2 pandemic (31), researchers suggest that pet animals may be susceptible to SARS-CoV-2 infection. Therefore, studies are recommended to verify which other pets and companion animals can be infected by SARS-CoV-2 (60). It is also recommended to limit the contact between the infected owner and pets, avoid kissing animals, apply basic hygiene measures (36, 58), and prevent pets from interacting with other animals or individuals outdoors (57). Even though CDC does not recommend routine testing of animals for SARS-CoV-2 (57), pet animals exposed to SARS-CoV-2 infected patients and presented clinical signs of new illness should be screened for SARS-CoV-2 as a precautionary measure (31, 57, 92). Moreover, general hygiene measures should be done after contact with animals and animal products, such as regular handwashing with soap (76).

### One Health Approach

The One Health approach includes joint planning and collaborative efforts of various sectors and disciplines that work together from local to global levels to maintain optimal health and welfare of people, animals, and plants in shared environments (105–108). Therefore, a thorough knowledge of the relationships between pathogen, native hosts, intermediate hosts, and environment together with the tendency of mutations, characteristics of animals-to-humans, and humans-to-humans transmission is crucial to understand the mechanism of COVID-19 and combat its spread (107, 109–113). Knowledge of the animal reservoir and the transmission cycle will help to prevent and mitigate the transmission of the virus (62, 110). The spread of the emerging SARS-CoV-2 can be controlled through rapid laboratory diagnosis, proper isolation, quarantine measures, developing effective vaccines and therapeutics (18, 109, 113, 114). In addition to surveillance of susceptible animals in close contact to humans, zoo animals, and wildlife animal species to highlight their role as an intermediate host or virus carrier (33, 61, 113, 115). It is crucial to establish an international cooperation sharing pathogen sequence libraries and updated databases to facilitate efforts in disease diagnosis and vaccine development (107). Such strategies will help to prevent and mitigate outbreaks of SARS-CoV-2 in these animals and the potential of spillback to humans (23). On the other hand, it is recommended to apply public health and biosecurity measures for workers in the field of meat and poultry processing for their protection, enhancement of food safety, and preservation of processing facilities during the pandemic (33, 116).

### Standard Precautions and Measures

Standard precautionary measures should be practiced at all times to maintain good personal and environmental hygiene. These measures include regular washing of hands with water and soap/detergent, using a disinfectant, wearing masks, avoid close contact with affected individuals, avoid crowded places, cover the mouth with tissue during sneezing or coughing, avoid touching the eye and nose, food hygiene practice, and thorough cooking of animal-based food items (115, 117–119).

## Concluding Remarks

This review provides an overview of the current knowledge of the relation between SARS-CoV-2 infection and the role played by animals. It was speculated that SARS-CoV-2 was originated from bats and passes through an unknown intermediate host, which facilitated its transfer to humans. Although it is not confirmed that pet animals play a role in the spreading of the SARS-CoV-2 pandemic, several animal species either received natural infection upon contact with an infected individual or after experimental infection. Therefore, it is recommended to apply basic hygiene measures to limit the contact between infected patients and pets. Several animal models have been reported as candidates for assessing the efficacy and safety of antiviral drugs or testing experimental vaccines against SARS-CoV-2. These models include mouse models, either transgenic mice, and adenoviral-infected mice, hamster models, cat, ferret, and primate models. To prevent the spread of the virus, collaborative efforts of different disciplines, such as public health, veterinary medicine, environmental sciences, and social sciences are crucial as the One Health approach. These studies will help to understand the potential hosts of the virus, the mechanism of transmission, and vaccine development. Moreover, public health measures for workers dealing with animals and animal-by-products are recommended together with the application of standard hygienic measures. The current review provided an overview of the available animal models and highlighted the advantages and disadvantages of each model to help researchers in their future research. Among the potential animal models, rhesus macaques were more highlighted the symptoms of COVID-19 disease in humans.

## Author Contributions

All authors listed have made a substantial, direct and intellectual contribution to the work, and approved it for publication.

## Conflict of Interest

The authors declare that the research was conducted in the absence of any commercial or financial relationships that could be construed as a potential conflict of interest.
